# Supplementary material for: miR-193b-3p Promotes Proliferation of Goat Skeletal Muscle Satellite Cells through Activating IGF2BP1
Source: Int J Mol Sci. 2022 Dec 12;23(24):15760. doi: 10.3390/ijms232415760 (PMC9779864; doi:10.3390/ijms232415760)
Supplement: Supplementary file 1 [file ijms-23-15760-s001.zip › Supplementary Figures S1-S7.pdf]

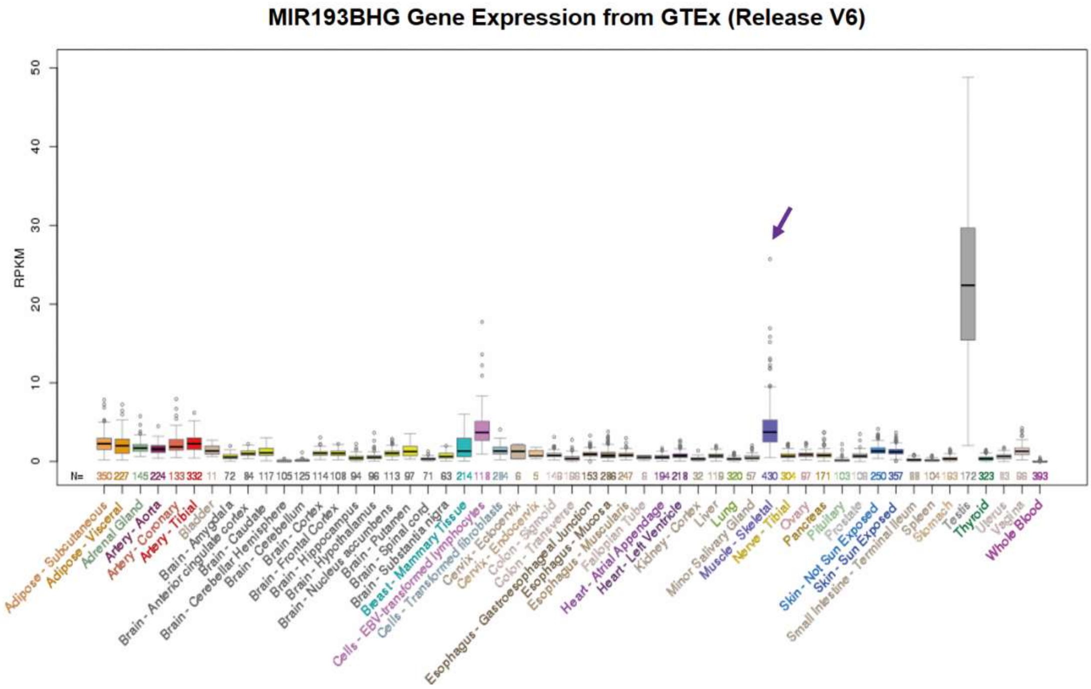

Supplementary Figure S1 MIR193BHG expression from 53 human tissues. Data are from GTEx (Release V6). A purple arrow marks the skeletal muscle.

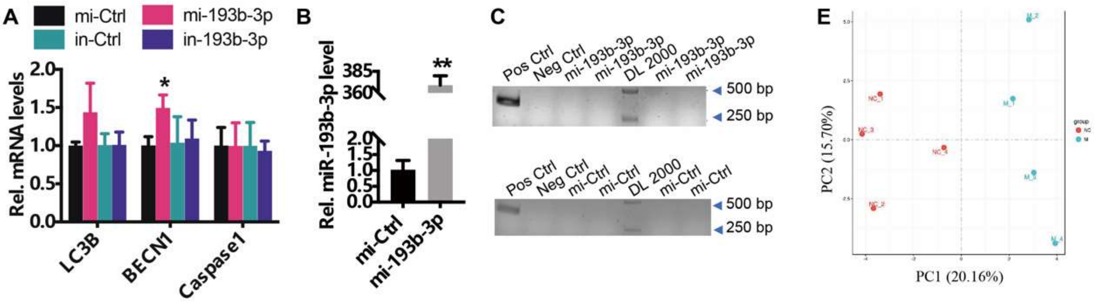

D

Summary of mRNA-seq data

Sample	Raw_Reads	Clean_Reads	Clean_Bases	Q20	Q30	GC(%)	Total_mapped Reads	Unique_mapped reads
mi-193b-3p_1	60,493,974	59,407,286	8.91G	97.73	93.72	51.85	57,709,519 (97.14%)	55,902,971 (94.10%)
mi-193b-3p_2	60,386,756	59,415,342	8.91G	97.87	94.02	52.07	57,799,628 (97.28%)	55,977,155 (94.21%)
mi-193b-3p_3	64,669,032	63,515,614	9.53G	97.90	94.04	51.82	61,846,484 (97.37%)	59,892,397 (94.30%)
mi-193b-3p_4	60,602,468	59,485,720	8.92G	97.87	94.01	51.72	57,840,582 (97.23%)	56,020,452 (94.17%)
mi-Ctrl_1	63,762,024	62,659,228	9.40G	97.80	93.89	52.05	60,895,959 (97.19%)	58,958,722 (94.09%)
mi-Ctrl_2	56,286,654	55,269,082	8.29G	97.82	93.95	51.84	53,725,761 (97.21%)	52,029,437 (94.14%)
mi-Ctrl_3	60,566,528	59,499,094	8.92G	97.77	93.85	52.03	57,815,425 (97.17%)	56,002,622 (94.12%)
mi-Ctrl_4	62,728,668	61,693,870	9.25G	97.67	93.60	51.83	59,897,877 (97.09%)	57,955,520 (93.94%)

Supplementary Figure S2 Basic information for mRNA transcriptome in miR-193b-3p-treated MuSCs.

(**A**) Effect of miR-193b-3p on the transcripts of genes related to apoptosis and autophagy. The unpaired two-tailed t-test was used to evaluate the means difference. Data are shown as mean ± MSE. * $P < 0.05$.

(B) mi-193b-3p levels quantified using qPCR.

(**C**) Mycoplasma contamination in culturing media of samples (mi-193b-3p and mi-Ctrl-treated) detected using polymerase chain reaction (PCR) and subsequent 1.5% agarose gels electrophoresis, with the positive control (Pos Ctrl), negative control (Neg Ctrl), and DNA ladder DL2000.

(**D**) The basic information for the poly^{A+} mRNA-seq output data.

(**E**) Transcriptome clustering based on principal component analysis (PCA). The red dot represents control (NC), and the green dot represents miR-193b-3p mimics treated sample.

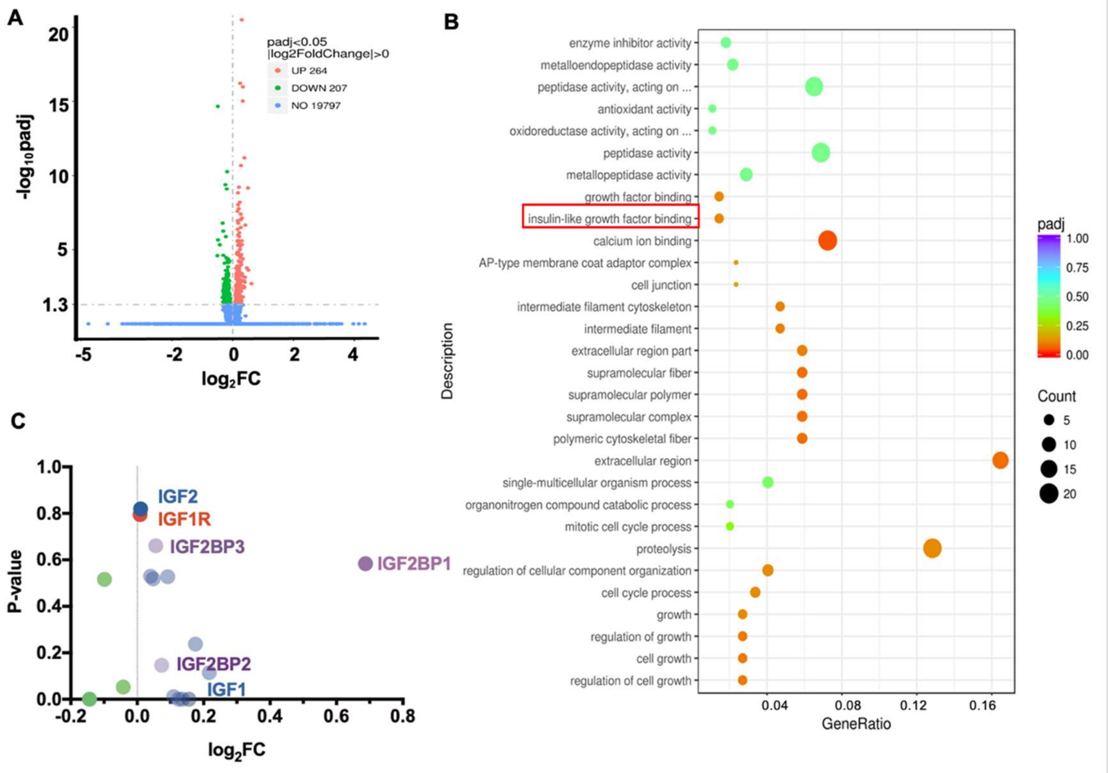

Supplementary Figure S3 Insulin-like growth factor 2 binding protein 1 (IGF2BP1) is the most miR-193b-3p-affected insulin genes.

(**A**) A volcano plot displays $\log_2$foldchange ($\log_2$FC) against $-\log_{10}p_{adj}$ from the t-test for all the transcript detected in mRNA-sequencing.

(**B**) Functional enrichment analyses of differential expression genes (DEGs) were implemented using the clusterProfiler R package (v. 3.4.4) with Padj < 0.05. A red box marks insulin-like growth factor binding (GO:0005520, Padj = 0.015).

(**C**) A volcano plot for the total 17 insulin family genes detected in RNA-seq. The green dot indicates that gene expression is decreased by miR-193b-3p mimics, while others are elevated compared to mimics control (mi-Ctrl).

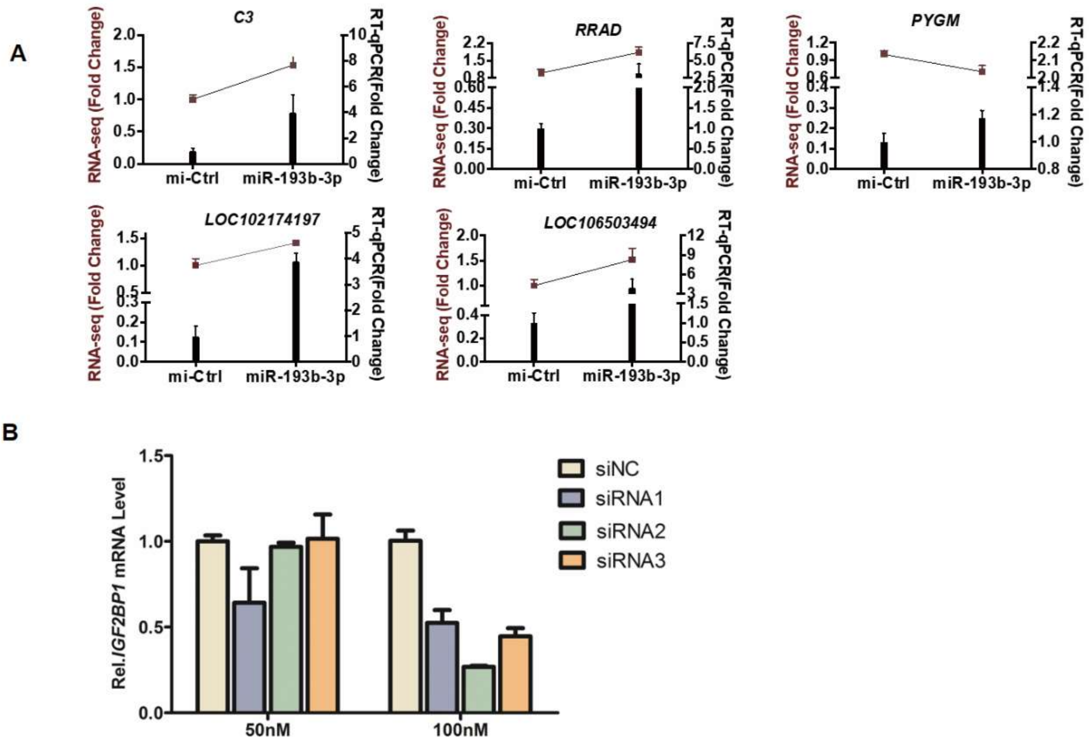

Supplementary Figure S4 qPCR validation of RNA-seq data (A), **efficiency of interfering RNAs against IGF2BP1(B).**

(**A**) Validation the abundance of the genes detected in RNA-seq. Data are shown as mean ± MSE. C3, Complement C3; RRAD, Ras Associated with Diabetes; PYGM, Glycogen Phosphorylase, Muscle Associated; LOC102174197, xaa-Pro aminopeptidase 2; LOC106503494, H1.2 linker histone.

(**B**) Efficiency of siRNAs against IGF2BP1 mRNA. IGF2BP1 abundance in at least three independent samples is quantified via RT-qPCR and calculated using the $2^{-\triangle\triangle Ct}$, with β-actin (ACTB) as an internal control and values of control (siNC) set to 1.

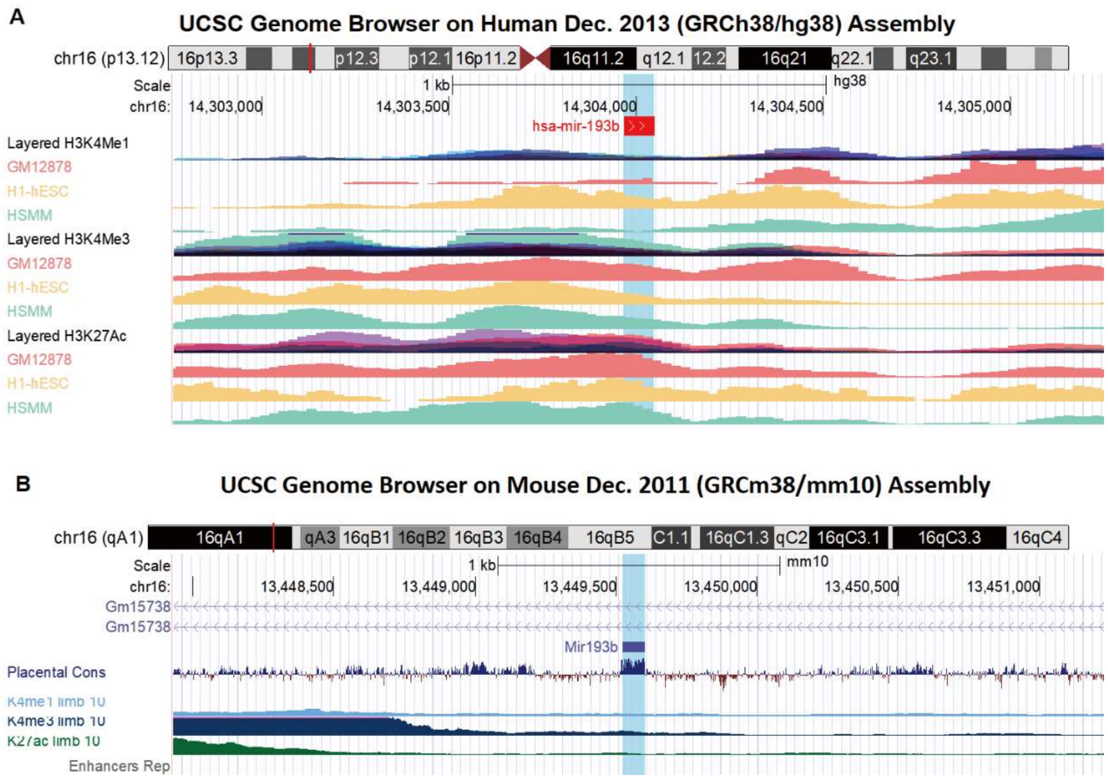

Supplementary Figure S5 Difference of histone modification between human and mouse mir193b loci.

(**A**) Hsa-mir-193b sits in the peak and valley of H3K4Me1/H3K4me3/ H3K27ac derived from 3 cell lines, including GM12878 (a lymphoblastoid cell line), H1-hESC (H1 human embryonic stem cells), and HSMM (human skeletal muscle myoblasts), in ENCODE UCSC, respectively.

(**B**) Histone modification of mmu-mir-193b gene region in mouse tissues in ENCODE UCSC.

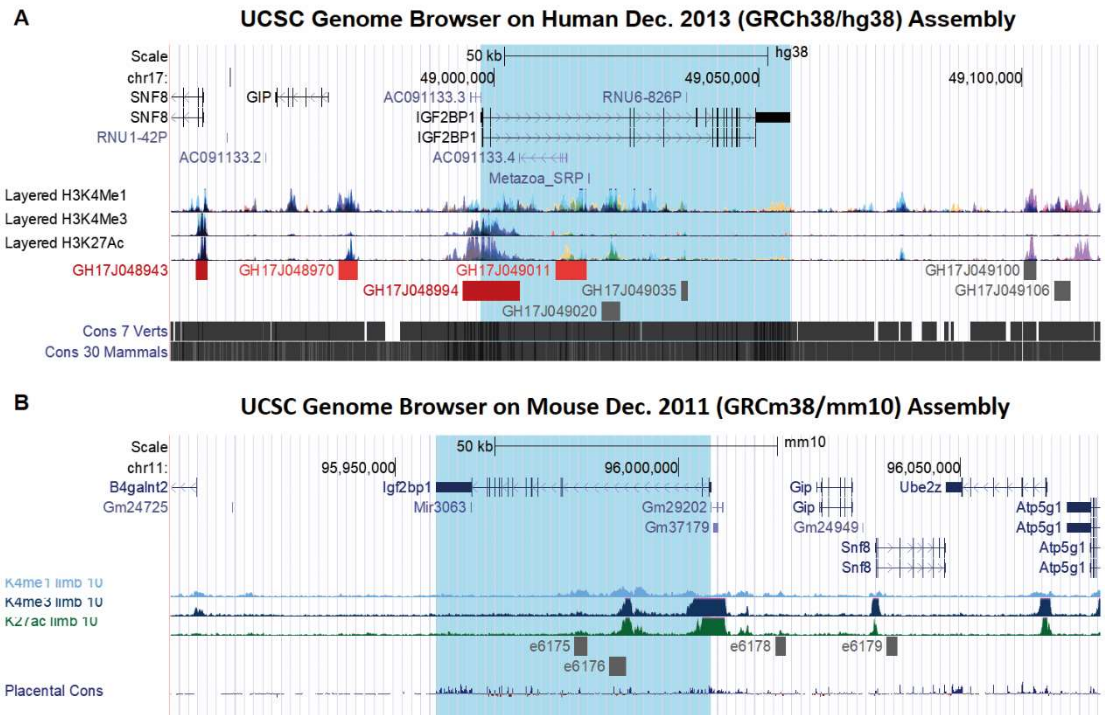

Supplementary Figure S6 Histone modification of human and mouse IGF2BP1 loci. Blue lines highlight IGF2BP1 gene region. Both human (**A**) and mouse (**B**) IGF2BP1 3′ UTR sits in the valley of H3K4Me3 and H3K27Ac.

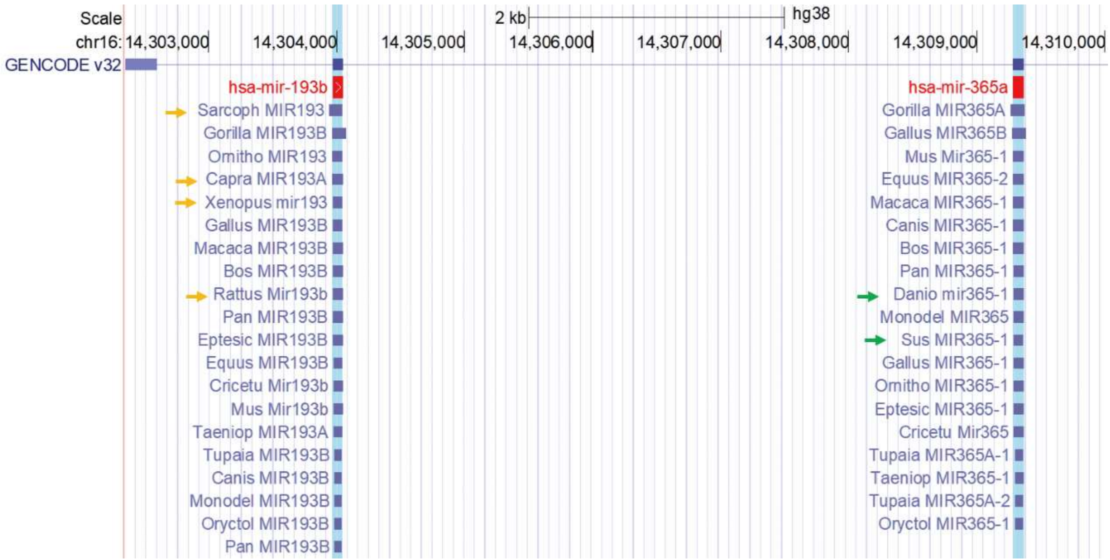

Supplementary Figure S7 Location of miR-193b and miR-365 is differed among species in UCSC. Blue lines highlight hsa-mir-193b and hsa-mir-365a. Yellow arrows mark animals containing miR193 conserve with hsa-mir-193b but lack miR365, while green arrows mark species containing conserve miR365 but lack miR193 on the same chromosome.
