# Supplementary material for: miR-193b-3p Promotes Proliferation of Goat Skeletal Muscle Satellite Cells through Activating IGF2BP1
Source: Int J Mol Sci. 2022 Dec 12;23(24):15760. doi: 10.3390/ijms232415760 (PMC9779864; doi:10.3390/ijms232415760)
Supplement: Supplementary file 1 [file ijms-23-15760-s001.zip › Supplementary Table S1.pdf]

Supplementary Table S1 Primers information for genes

Gene	Sequence of primer (5'-3')	Tm (°C)	Size (bp)
PGK1 （XM_005700601.3）	F: TGGACCTGTGGGTGTATT<br>R: CTGACTTTATCCTCCGTGTT	60.3	159
SDHA (XM_018065656.1)	F: GGAACATGGAGGAGGACAACT<br>R: CCAAAGGCACGCTGGTAGA	60.3	187
β-actin (NM_001314342.1)	F: GCGGCATTCACGAAACTAC<br>R: AGGGCAGTGATCTCTTTCT	63.3	145
IGF2BP1 (XM_013972119.2)	F: CCCAGTCCAAGATCGACGTA<br>R: CAGCCGTTTTGGTGTCCTTG	52.0	150
miR-193b-3p	F: AACTGGCCCACAAAGTCCCGCT	53.9	-
chi-MKL2 (XM_018040535.1)	F: AAGCACAAGATTCGGAGTC<br>R: TGGAGTCTACAGGAAGGATG	60.3	202
chi-PARN (XM_013974855.2)	F: GAATCACCATTACCACGCCAA<br>R: GTGTCAGAAATCTCCCCCGA	53.9	120
chi-BFAR (XM_005697517.3)	F: TCTACAGAGACAGGTTTTTAT<br>R: AGAGGACAGATGGTGTGAAT	58.3	316
mmu-β-actin (XM_030254057.1)	F: GGCTATGCTCTCCCTCACG<br>R: CACGCACGATTTCCCTCTC	55.9	130
mmu-GAPDH (XM_017321385.2)	F: AGGTTGTCTCCTGCGACTTCA<br>R: TGGTCCAGGGTTTCTTACTCC	62.0	294
mmu-Hprt (NM_013556.2)	F: GATCAGTCAACGGGGGACATA<br>R: ATCCAACACTTCGAGAGGTCC	62.0	199
mmu-IGF2BP1 *(NM_009951.4)*	F: GAAACACCTGACTCCAAAGTTC<br>R: GGTCTCTAGCTTTACTTCCTCC	62.4	138
mmu-MKL2 (NM_181860.1)	F: GGCTGCGTAAAAGAGGTGT<br>R: TTCCGTTCGTTCAGAGTGG	64.1	186
mmu-PARN (XM_006522652.3)	F: AAGAACGCAAAAGGAGAGAGC<br>R: GGGCAGTAGAACTGATGAATCG	60.3	169
mmu-BFAR (XM_021209265.2)	F: TTTGCTGAGAGATGGAGGAG<br>R: GTTCAAGGTGGTGGGGTTA	64.1	155
RRAD (XM_018062096.1)	F: CGAGTGCGTTTACAAGGTGC<br>R: CCCATCCTGCTCCCAAATGT	65.0	187
C3 (XM_018050944.1)	F: ATCCCCTGACCATCACCGTA<br>R: GAGTTCCCCTGCGTGTTGTA	59.5	103
LOC106503494 (XM_018038510.1)	F: GCACCCAAGAAGAAGTAGGTTG<br>R: TTAGGTAGTGCCAGCCATCT	60.3	190
LOC102174197 (XM_018045398.1)	F: GGGGCAGAACAACTGGAGAA<br>R: TCTTTGGTGGGGCTGTAGTG	60.3	119
